# Diabetes, periodontal disease, and novel therapeutic approaches- host modulation therapy

**DOI:** 10.3389/fcdhc.2025.1529086

**Published:** 2025-03-03

**Authors:** Ying Gu, Lorne M. Golub, Hsi-Ming Lee, Stephen G. Walker

**Affiliations:** ^1^ Department of General Dentistry, School of Dental Medicine, Stony Brook University, Stony Brook, NY, United States; ^2^ Department of Oral Biology and Pathology, School of Dental Medicine, Stony Brook University, Stony Brook, NY, United States

**Keywords:** diabetes, oral diseases, host modulation therapy, periodontitis, therapeutics

## Abstract

Diabetes mellitus is a much-studied disorder, characterized by hyperglycemia and numerous oral and medical complications. The latter includes (above all) decreased life-span — and these are widely discussed in the dental and medical literature. The oral complications include impaired wound healing; increased severity of periodontal disease and peri-implantitis; dry mouth (xerostomia); and dental caries. The relationship between diabetes and oral health is bi-directional: Optimal management of local oral disease can profoundly affect the systemic metabolic control of the diabetic patient, and strict management of the patient’s hyperglycemia can reduce its impact on oral disease. The only host modulation therapy (HMT), approved by the U.S. Food and Drug Administration (FDA) to treat periodontal disease, is a novel NON-antimicrobial (low-dose) formulation of doxycycline (Periostat^®^; 20 mg b.i.d). A publication in Scientific Reports (2017), which supported the clinical rationale of efficacy and safety of low-dose doxycycline in diabetics, stated: “doxycycline not only ameliorated insulin resistance, fasting blood glucose, and insulin levels, and lipid profiles in the circulation and liver, but also improved islet morphology and increased glucose-stimulated insulin secretion.” Additional developments include the biphenolic chemically-modified curcumins, as HMT for managing oral diseases. A lead compound, chemically-modified curcumin 2.24 (CMC2.24), has demonstrated safety and efficacy *in vitro*, in cell culture, and *in vivo* using mouse, rat, rabbit, and dog models of disease. In conclusion, novel host-modulation compounds have shown significant promise as adjuncts to traditional local therapy in the clinical management of periodontal and other oral diseases.

## Introduction

Diabetes mellitus is an all-too common carbohydrate metabolism disorder, characterized by elevated blood glucose levels and the formation of advanced glycation end products (AGEs) (1). AGEs interact with their cellular receptors RAGEs (receptors for advanced glycation end products) on inflammatory cells (and other cells), which leads to a chronic inflammatory condition and subsequent medical complications (1, 2). These widely recognized medical complications include cardiovascular disease, impaired wound healing (which when severe could result in limb amputation), retinopathy, and nephropathy. Other well-known complications that have been reported include excessive urinary protein excretion, cataract development in the eye, and reduced life expectancy (3, 4). The oral complications related to diabetes include xerostomia, dental caries, gingivitis, periodontal disease, tooth loss, increased tendency to oral infections, burning mouth, taste disturbance, and poor oral wound healing (3, 5). As a result, periodontal disease has now been recognized as a significant complication of diabetes (1–3, 5). Oral and systemic conditions impact each other. This warrants a comprehensive treatment approach that should involve both medical and oral health care providers, to better manage the glycemic control and its multiple complications in these patients.

## Diabetes and periodontal disease

As discussed above, periodontal disease has long been recognized as an important oral complication associated with diabetes. As described in two reports in The Journal of the American Dental Association (JADA) (2016, 2022) (1, 2), the relationship between diabetes and periodontitis is bi-directional; i.e., regarding diabetic patients, “some people with periodontitis (severe gum disease) have a harder time keeping their blood sugar levels under control, whereas other people whose blood sugar is not well controlled, may have trouble with gum disease.”

The extensive list of studies by Golub & colleagues on the impact of diabetes on periodontal disease was largely conducted with an animal model of Type I diabetes and these are addressed below. However, Type 2 diabetes mellitus (T2DM) is more common, and has been described “as a new epidemic.” (5) In an extensive study on more than 60,000 “senior women (mean age 70 years) almost 5% had T2DM and gingivitis and periodontitis.” (3) Moreover, in a review published 25 years ago by Ryan, Ramamurthy, Sorsa & Golub (1999) (4), both type I and type II diabetes models were reported to be “associated with unusually aggressive periodontitis.”

H. Loe (1993) (6) proposed that periodontal disease should be described as “the sixth complication of diabetes mellitus,” and Preshaw et al. (2012) (7) proposed that achieving periodontal health should be promoted as an “integral component of diabetes management.” Clearly, the link between diabetes and oral health highlights the need for effective treatment of both the systemic (diabetes) and local (periodontitis) diseases for optimal care of these patients.

Optimal local therapy (oral hygiene instruction and scaling & root planing; SRP) to control/decrease periodontitis is essential in all patients, but especially in diabetics. Addressing mechanisms, Golub et al. (1983) (4, 8, 9) were the 1^st^ to demonstrate that diabetes (in experimental rat models) upregulates the production of host-derived collagenolytic matrix metalloproteinases (MMPs) in gingiva. Extraoral tissues (skin) were studied later with similar results (see below). In the early (1980’s) studies on diabetes in rats (8–10), it was suggested that an overgrowth of anaerobic microorganisms, due to impaired blood supply in the gingiva, was the cause of increased host-derived MMPs. Therefore, tetracyclines (TCs) were administered to suppress these anaerobes and reduce microbial endotoxin (lipopolysaccharide; LPS) load, thus reducing MMP production and activation by the host tissues. However, when the studies were repeated using germ-free rats which were rendered diabetic, again the MMPs were pathologically elevated, and again TCs (minocycline, doxycycline) were found to suppress the MMPs. This novel observation was clearly independent of the antimicrobial efficacy of these antibiotics!

**Figure 1:**
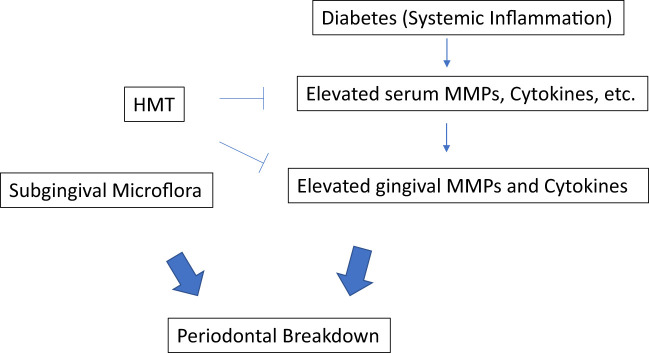
Host modulation therapy.

Based on the above studies on the gingiva in germ-free rats, and on skin (9), it was recognized that the increase of MMPs produced during diabetes was due to an abnormal host-response, and not due to an altered subgingival microflora. Also, the ability of TCs to suppress the excess MMPs was clearly independent of the antibiotic activity of the tetracyclines and was due to the unexpected ability of these antibiotics to beneficially modulate the host response! Additional research expanded this novel concept, which was now called “Host-Modulation therapy (HMT).” There are different types of HMT agents have been investigated in treating the periodontal disease (11), mainly: 1. MMP inhibitors such as TCs (Periostat) to reduce the excessive host-derived MMP levels; 2. anti-inflammatory drugs such as NSAIDs to lower excessive levels of cytokines and prostanoids, therefore blocking the tissue degradation; and 3. bone remodeling modulation drugs such as bisphosphonates to reduce bone resorption by modulating osteoclast function (**Figure 1**). In this mini review, we will focus on the MMP inhibitors as the HMT. Intense research has been conducted on the NON-antibiotic efficacy of tetracyclines, particularly minocycline, and later, doxycycline (8). Since doxycycline was more effective than the others, this compound was selected for further study - - its dose (per capsule) as a “host-modulator” was progressively reduced, from the traditional antimicrobial 50-100 mg/day regimens, to prevent antibiotic side-effects (e.g., emergence of antibiotic-resistant bacteria). Eventually, after numerous clinical studies, a dose of 20 mg doxycycline, b.i.d., was found to be “safe and effective” - - this dose was too low to function as an antibiotic, but did suppress pathologically-elevated MMPs, as well as inflammatory mediators (Prostaglandin E_2_, cytokines, etc.) (8). Long-term *in vivo* studies in animals, & subsequent numerous clinical trials demonstrating safety and efficacy, resulted in this novel NON-antimicrobial formulation receiving U.S. government, Food and Drug Administration (FDA)-approval as Periostat^®^ in 1998 (8). Subsequently, a sustained-release once per day version of nonantimicrobial-dose doxycycline (which would promote long-term compliance), called Oracea^®^ (40 mg q.d.), was developed. This formulation was developed for the treatment of the common chronic inflammatory skin diseases, acne and rosacea. After numerous clinical trials on patients with rosacea, Oracea^®^ was approved by the FDA in 2006 (8). Although, currently, Oracea^®^ has not yet been tested in diabetic humans suffering with periodontitis (or other diseases), it presumably could be more effective than Periostat^®^ since long-term compliance would be improved by the once-per-day formulation compared to b.i.d. for Periostat. A list of clinical studies demonstrating the efficacy of these NON-antibiotic dose doxycycline regimens on patients with various inflammatory/collagenolytic diseases includes rheumatoid arthritis, post-menopausal osteopenia, sterile corneal ulcers, epidermolysis bullosa, and acute coronary syndrome (see review by Golub and Lee (2020) (8).

Additional host modulation therapeutic (MMP-Inhibitor) regimens are currently being developed (see below) in collaboration with Dr. Francis Johnson, Professor of Chemistry and Pharmacology at Stony Brook University. These are based on novel chemical modifications of the natural spice, curcumin. A lead compound has emerged called CMC2.24 which was recently discussed by Golub and Lee (8).

## Diabetes and peri-implantitis

Diabetes mellitus has been associated with an increased risk of peri-implantitis, a condition characterized by inflammation and loss of supporting tissues around dental implants. This relationship is attributed to several factors, including impaired wound healing, altered immune response, and increased susceptibility to infections in diabetic patients (12, 13) Studies have shown that individuals with poorly controlled diabetes are more likely to experience peri-implant diseases compared to non-diabetic individuals, emphasizing the need for optimal management of diabetes in patients undergoing dental implant therapy (14, 15).

Of interest, Dr. Joseph Bacigalupo, a private practitioner, recently reported his extensive clinical experience on treating his periodontitis and peri-implantitis patients using adjunctive NON-antibiotic-dose doxycycline (Periostat^®^) over many years. The periodontal and peri-implant tissues continue to appear healthy and stable in about 800 patients (most of these patients with periodontitis; others with peri-implantitis) who have been or still are being medicated (adjunctively) with Periostat^®^ (16). There has been no significant adverse reactions to this novel HMT, seen to date, in this large group of patients. Moreover, based on routine medical examinations, no issues have been raised by the patients’ physicians.

## Diabetes and dental caries

Individuals with diabetes are also at a higher risk for developing dental caries due to factors such as hyperglycemia, which can lead to dry mouth and decreased salivary flow, and increased glucose levels in saliva, impairing the mouth’s ability to neutralize acids and wash away food particles (17). Moreover, diabetes can alter the composition of the oral microbiota, promoting an environment conducive to caries development (18). Conversely, the presence of dental caries can complicate diabetes management, as infections can lead to elevated blood glucose levels (5). Thus, the correlation between diabetes and dental caries (as in diabetic patients with periodontitis) is bidirectional; as discussed earlier, one can exacerbate the other. Clearly, maintaining optimal oral health is crucial for individuals with diabetes to prevent medical complications and to improve overall health outcomes.

Current non-invasive, non-surgical approaches to manage dental caries are very limited other than fluoride treatment and promoting optimal oral hygiene. Most current therapies primarily focus on the surgical/mechanical management of dental caries, which is time-consuming, uncomfortable, and expensive. Preliminary research (19, 20) carried out in our lab, recently demonstrated that doxycycline and chemically-modified tetracycline can effectively reduce the biodegradation of dental composite materials, thus preventing the separation of the tooth-restoration interface. This could reduce the risk of developing secondary dental caries. Ongoing and future research with a novel HMT compound not categorized as a tetracycline, i.e., CMC2.24, is discussed below.

## Future host-modulation agents: the chemically-modified curcumins

**Figure 2:**
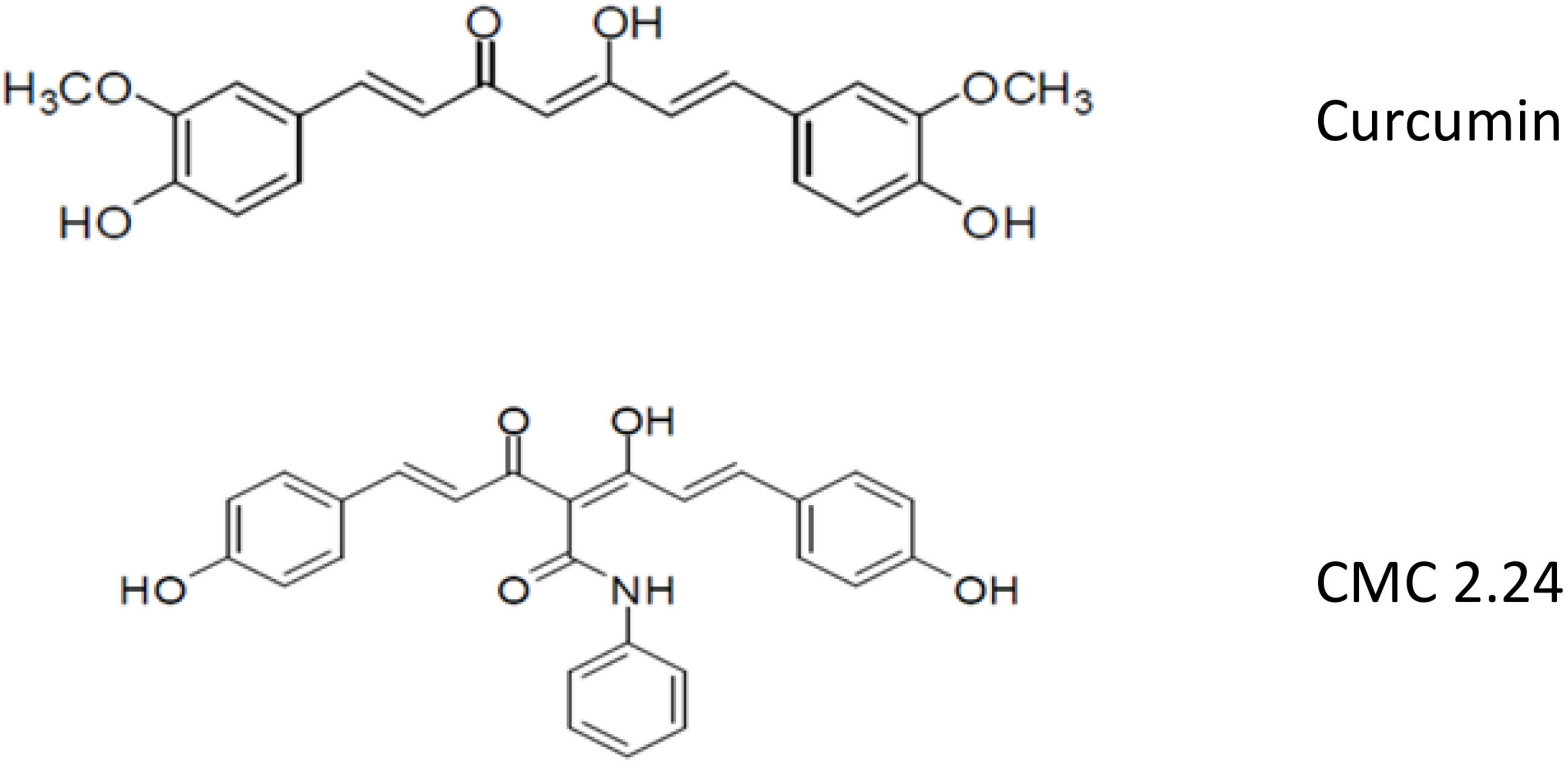
Structures of Curcumin and CMC 2.24.

Although the discovery of the unexpected property of tetracyclines as MMP-inhibitors has been translated into novel first generation (Periostat^®^, Oracea^®^) and second generation (CMT-3) pharmaceutical agents, yet a third-generation of host-modulating therapeutics is now being developed (**Figure 2**). Based on our previous identification of the MMP-inhibiting, active-site on the 4-phenolic-ring tetracyclines (21–23), we began to focus on di-phenolic compounds with the same cation (Zn^++^, Ca^++^) -binding site, the β-diketone moiety, namely the bis-aroyl methanes and curcumins. The bis-aroyl methane compounds exhibited positive, but weak MMP-inhibitory efficacy *in vitro*, i.e., high IC_50_ (µM) values, and thus were quickly abandoned. The focus then shifted to curcumin, a dietary herbal ingredient derived from turmeric, which historically has been advocated as a safe and effective treatment for a variety of diseases (24–26). However, this compound’s insolubility and poor absorption by the oral route has limited its clinical applications (27).

To address the therapeutic potential of this well-known herbal supplement, our Stony Brook dental school team has been collaborating with Professor Francis Johnson, PhD (in the Departments of Chemistry and Pharmacology at Stony Brook University) who has synthesized, developed, and tested a series of novel chemically-modified curcumins (CMCs) with various side-chains added to the carbon-4 position of this bi-phenolic compound (**Figure 2**). Based on these studies, a lead tri-phenolic compound was identified, a tri-ketonic phenylaminocarbonyl curcumin (CMC2.24), and has been tested *in vitro*, in cell and tissue culture, and *in vivo* using various animal species including mice, rats, rabbits and, most recently, dogs (24–26, 28–30). In addition to CMC2.24 exhibiting superior efficacy compared to curcumin, as an inhibitor of inflammatory mediators and MMPs, this novel compound was found to be even more effective in reducing alveolar bone loss *in vivo.* In stark contrast, the natural curcumin had NO effect on alveolar bone loss (29, 30). CMC2.24 decreased the number of osteoclasts in alveolar bone characterized immunohistochemically as TRAP-positive and caspase-3 positive-cells (28–30). It also suppressed the pro-inflammatory cytokine, InterleukinIL-1β (IL-1β), and reduced levels of pro- and activated forms of leukocyte-type gelatinase (MMP-9) in the gingival tissues, as well as reducing systemic biomarkers, i.e., IL-1β and the activated form of MMP-8 (aMMP-8; leukocyte-type collagenase) in the circulation. Our lead compound as a host-modulator, CMC2.24, orally administered to two rat models of disease [the locally-induced (microbial endotoxin/LPS) disease, periodontitis, and the systemic disease, diabetes], resulted in a marked reduction of inflammatory cytokines and MMPs in the gingival tissues, decreased bone loss, and decreased activation of p65 Nuclear factor-kappa B (NFκB) and p38 Mitogen-activated protein kinase (MAPK) (28). CMC2.24 administration did not reduce the severe hyperglycemia (at least during the 1-month experimental protocol), yet completely prevented alveolar bone loss which was induced in these severely diabetic rats by multiple LPS injections into the gingiva. Addressing causality of this decreased periodontal breakdown, this novel therapeutic compound also suppressed the excessive levels of pro- and activated MMP-2 and MMP-9 (gelatinases/type IV collagenases) in the diseased gingival tissues, as well as the activated (lower molecular weight) form of the dominant collagenase, MMP-8, in these oral tissues. The dominant cytokines in the inflamed gingiva, IL-1β and Interleukin-6 (IL-6) (Tumor necrosis factor alpha (TNF-α) was not detected), were also reduced locally by this treatment, and similar beneficial biomarker changes were seen systemically in the circulation as well as in skin (24, 28–30).

Of particular relevance to an all-too-common medical complication in diabetics, i.e., impaired healing of skin wounds, the collagen atrophy and excessive collagen crosslinking (i.e., “leathery-like” texture) were also “normalized” in the CMC2.24-treated diabetic animals (24). An additional mechanism was its ability to normalize a key resolvin (31, 32) (ResolvinD_1_; a docosahexanoic acid derivative) and interleukin-10 (an anti-inflammatory cytokine), which are both under-expressed in macrophages isolated from severely hyperglycemic diabetic rats (33). The same research team showed that CMC2.24 significantly suppressed the pro-inflammatory M1 phenotype of macrophages while promoting the pro-resolving M2 phenotype, an effect which is essential in the wound healing process (34). To further address the potential utility of this novel compound, a recent publication by Gnocchi et al. (2024) (35) described the recent novel process of zein spray-dried biocompatible microparticles loaded with CMC2.24. The results indicated that delivering the compound with this novel topical delivery system reduced intracellular oxidative stress, which is an important factor in promoting the healing of chronic wounds which can be severely compromised in diabetic patients (35).

In addition, CMC 2.24 showed promising therapeutic potential in preventing recurrent dental caries based on *in vitro* studies. The compound effectively reduced composite resin biodegradation caused by the cariogenic pathogen *Streptococcus mutans* (19). This would be expected to decrease recurrent caries by stabilizing the interface between the dental material and the tooth structure. Future studies will evaluate the effectiveness of CMC 2.24 in preventing the process of recurrent dental caries in bovine teeth and in diabetic animal models.

In conclusion, novel host-modulation compounds described in this mini review, including those already clinically available (Periostat^®^ and Oracea^®^), and those currently under development (especially CMC2.24), have shown significant efficacy in reducing MMP and inflammatory cytokine levels, therefore inhibiting soft and hard tissue destruction in periodontitis and peri-implantitis. In addition, they have also demonstrated significant efficacy in reducing the composite biodegradation; thus reducing the risk of developing secondary caries. Overall, these compounds showed great promise as adjuncts to traditional local therapy in the clinical management of periodontitis and other oral and systemic diseases.
